# Supplementary material for: An operator-based approach to topological photonics
Source: Nanophotonics. 2022 Nov 14;11(21):4765–80. doi: 10.1515/nanoph-2022-0547 (PMC11501349; doi:10.1515/nanoph-2022-0547)
Supplement: Supplementary file 1 — Supplementary Material Details [file j_nanoph-2022-0547_suppl.pdf]

Supplemental Material: An operator-based approach to topological photonics

Alexander Cerjan[1,*] and Terry A. Loring[2,†]
[1] Center for Integrated Nanotechnologies, Sandia National Laboratories, Albuquerque, New Mexico 87185, USA
[2] Department of Mathematics and Statistics, University of New Mexico, Albuquerque, New Mexico 87131, USA
(Dated: September 7, 2022)

MATRIX SQUARE ROOTS AND SYMMETRIES

In the main text we relied on the fact that if a positive semi-definite matrix M satisfies a symmetry then so must $M^{\frac{1}{2}}$ and $M^{-\frac{1}{2}}$. Indeed, this claim is also true for bounded operators on a Hilbert space. Note that in operator theory [1], the term positive is more common than positive semi-definite. We will prove things for the finite matrix case, where eigenvalues are guaranteed and the spectral theorem is simpler to state.

If M is positive semidefinite, then by the spectral theorem we can factor M as

$$M = UDU^{\dagger} \tag{S1}$$

with U unitary and D diagonal with the diagonal elements $\lambda_j = D_{j,j}$ all in the closed, finite interval $[0, \|D\|]$.

Given a positive semidefinite matrix M there will be a unique positive semidefinite matrix N so that $N^2 = M$. Of all the many square roots of M, it is only N that is anointed with the notation $M^{\frac{1}{2}}$. A good way to understand N is via Eq. (S1). Given that factorization, one quickly finds (see [2, Ex. 2.16.]

$$M^{\frac{1}{2}} = UD_1U^{\dagger} \tag{S2}$$

where D_1 is again diagonal, but with jth diagonal element $\sqrt{\lambda_j}$. This way of calculating the square root makes it hard to track the effect of a symmetry, so we look to one of the many alternate means of calculating M by hand. We are not discussing numerical methods of computing matrix square roots that respect symmetries, but that is of interest [3, § IV].

Given a sequence of polynomials p_n, if we select this so that we have good convergence $p_n(\lambda) \to \sqrt{\lambda}$ then we will have

$$M^{\frac{1}{2}} = \lim_{n\to\infty} p_n(M). \tag{S3}$$

To be technical, we are applying the Weierstrass Approximation Theorem [2, § 6.2.1] which guarantees a sequence of polynomials that uniformly converge on the interval $[0, \|M\|]$. The advantage of this approach is that with a polynomial $p(\lambda) = \sum a_n \lambda^n$ we need not use Eq. (S2). Instead, we can work with the more direct formula

$$p(M) = \sum a_n M^n. \tag{S4}$$

We get now to our first result. It is not new, but in the math literature one will generally not see any antiunitary operators, and will instead find results about elements of real C^*-algebras [4]. As such, it is simpler to provide direct proofs rather than to translate between the two pictures.

Lemma 1. *Suppose $\mathcal{U} : \mathbb{C}^n \to \mathbb{C}^n$ is a unitary or antiunitary operator. Suppose also that M is a positive semidefinite n-by-n matrix that we treat as a linear operator on $\mathbb{C}^n$. If $\mathcal{U}$ commutes with M then $\mathcal{U}$ commutes with $M^{\frac{1}{2}}$.*

Proof. We are assuming $\mathcal{U} \circ M = M \circ \mathcal{U}$ and it immediately follows that

$$\begin{aligned}\mathcal{U} \circ M \circ \cdots \circ M &= M \circ \mathcal{U} \circ \cdots \circ M \\ &\vdots \\ &= M \circ \cdots \circ M \circ \mathcal{U}\end{aligned}$$

which says $\mathcal{U} \circ M^n = M^n \circ \mathcal{U}$. For the case of unitary $\mathcal{U}$, everything proceeds as expected,

$$\mathcal{U} \circ \left(\sum a_n M^n\right) = \left(\sum a_n M^n\right) \circ \mathcal{U}. \tag{S5}$$

Unfortunately, for antiunitary $\mathcal{U}$ we find

$$\mathcal{U} \circ \left(\sum a_n M^n\right) = \left(\sum \overline{a_n} M^n\right) \circ \mathcal{U}. \tag{S6}$$

However, fortunately, we are able to select the p_n to have only real coefficients, by the the original theorem of Weierstrass [5], so we find $\mathcal{U} \circ p_n(M) = p_n(M) \circ \mathcal{U}$ for both the unitary and antiunitary cases. Finally, both unitary and antiunitary operators are continuous, so we pass to the limit and are done by Eq. (S3) □

To be fair, we are jumping over some complications when we say we are passing to the limit. What we need is at least point-wise convergence for

$$\lim_{n\to\infty} (\mathcal{U} \circ p_n(M)) = \mathcal{U} \circ \left(\lim_{n\to\infty} p_n(M)\right)$$

and

$$\lim_{n\to\infty} (p_n(M) \circ \mathcal{U}) = \left(\lim_{n\to\infty} p_n(M)\right) \circ \mathcal{U}.$$

In the unitary case these are standard results. The antiunitary versions have simple, but not short, proofs. At the core of these proofs is the fact that the antiunitary property

$$\langle \mathcal{U}(\boldsymbol{v}), \mathcal{U}(\boldsymbol{w})\rangle = \overline{\langle \boldsymbol{v}, \boldsymbol{w}\rangle}$$

* awcerja@sandia.gov
† loring@math.unm.edu

implies that $\mathcal{U}$ preserves distances:

$$\|\mathcal{U}(\boldsymbol{x})-\mathcal{U}(\boldsymbol{y})\|=\|\boldsymbol{x}-\boldsymbol{y}\|.$$

Lemma 2. *Suppose $\mathcal{U}:\mathbb{C}^n\to\mathbb{C}^n$ is a unitary or antiunitary operator. Suppose also that M is a positive semidefinite n-by-n matrix. If $\mathcal{U}$ anti-commutes with M then $\mathcal{U}$ anti-commutes with $M^{\frac{1}{2}}$.*

Proof. The proof is essentially as before, except that we can only prove that $\mathcal{U}\circ M^n=-M^n\circ\mathcal{U}$ for odd values of n. However, we can approximate the odd function

$$\lambda\mapsto\begin{cases}\sqrt{\lambda} & \text{for } \lambda\geq 0\\ -\sqrt{-\lambda} & \text{for } \lambda\leq 0\end{cases}\tag{S7}$$

on the larger interval $[-\|M\|,\|M\|]$ by polynomials, and this means we can zero out all the coefficients in even positions. The proof then proceeds as before. □

Lemma 3. *Suppose $\mathcal{U}:\mathbb{C}^n\to\mathbb{C}^n$ is a unitary or antiunitary operator. Suppose also that M is an invertible n-by-n matrix. If $\mathcal{U}$ commutes with M then $\mathcal{U}$ commutes with M^{-1}.*

Proof.

$$M\circ\mathcal{U}=\mathcal{U}\circ M\circ\mathcal{U}\circ M^{-1}\tag{S8}$$

$$M^{-1}\circ M\circ\mathcal{U}\circ M^{-1}=M^{-1}\circ\mathcal{U}\circ M\circ M^{-1}\tag{S9}$$

which simplifies to

$$\mathcal{U}\circ M^{-1}=M^{-1}\circ\mathcal{U}\tag{S10}$$

□

The proof is easily adapted to prove the following.

Lemma 4. *Suppose $\mathcal{U}:\mathbb{C}^n\to\mathbb{C}^n$ is a unitary or antiunitary operator. Suppose also that M is an invertible n-by-n matrix. If $\mathcal{U}$ anti-commutes with M then $\mathcal{U}$ anti-commutes with M^{-1}.*

[1] J. B. Conway, *A course in operator theory*, Graduate Studies in Mathematics, Vol. 21 (American Mathematical Society, Providence, RI, 2000) pp. xvi+372.

[2] M. T. Vaughn, *Introduction to mathematical physics* (Wiley-VCH Verlag GmbH & Co. KGaA, Weinheim, 2007) pp. xiv+527.

[3] T. A. Loring and F. Vides, Computing Floquet Hamiltonians with symmetries, J. Math. Phys. **61**, 113501, 23 (2020).

[4] H. Schulz-Baldes and T. Stoiber, Invariants of disordered semimetals via the spectral localizer, EPL (Europhysics Letters) (2021).

[5] K. Weierstrass, Über die analytische darstellbarkeit sogenannter willkürlicher functionen einer reellen veränderlichen, Sitzungsberichte der Königlich Preußischen Akademie der Wissenschaften zu Berlin **2**, 633 (1885).
